# Identification of Biomarkers Based on Differentially Expressed Genes in Papillary Thyroid Carcinoma

**DOI:** 10.1038/s41598-018-28299-9

**Published:** 2018-07-02

**Authors:** Jun Han, Meijun Chen, Yihan Wang, Boxuan Gong, Tianwei Zhuang, Lingyu Liang, Hong Qiao

**Affiliations:** 1 Department of Endoerinology and Metabolism, The Second Affiliated Hospital, Harbin Medical University, Harbin, 150001 China; 2 College of Bioinformatics Science and Technology, Harbin Medical University, Harbin, 150081 China; 3 Faculty of Vehicle Engineering and Mechanics, Dalian University of Technology, Dalian, 116024 China; 4 Department of Endoerinology and Metabolism, Mu danjiang Medical University Affiliated Hongqi Hospital, Mu danjiang, 157000 China; 5 Internal medicine, Hebei Provincial Eye Hospital, Xingtai, Hebei 054001 China

## Abstract

The incidence of papillary thyroid carcinoma (PTC) is increasing rapidly throughout the world. Hence, there is an urgent need for identifying more specific and sensitive biomarkers to explorate the pathogenesis of PTC. In this study, three pairs of stage I PTC tissues and matched normal adjacent tissues were sequenced by RNA-Seq, and 719 differentially expressed genes (DEGs) were screened. KEGG pathway enrichment analyses indicated that the DEGs were significantly enriched in 28 pathways. A total of 18 nodes consisting of 20 DEGs were identified in the top 10% of KEGG integrated networks. The functions of DEGs were further analysed by GO. The 13 selected genes were confirmed by qRT-PCR in 16 stage I PTC patients and by The Cancer Genome Atlas (TCGA) database. The relationship interactions between DEGs were analysed by protein-protein interaction networks and chromosome localizations. Finally, four newly discovered genes, *COMP*, *COL3A1*, *ZAP70*, and *CD247*, were found to be related with PTC clinical phenotypes, and were confirmed by Spearman’s correlation analyses in TCGA database. These four DEGs might be promising biomarkers for early-stage PTC, and provide an experimental foundation for further exploration of the pathogenesis of early-stage PTC.

## Introduction

Thyroid carcinoma is the most common malignancy in the endocrine system. Papillary thyroid carcinoma (PTC) is the most common pathological type of thyroid carcinoma, accounting for approximately 80% of all thyroid carcinomas^1^. Its incidence is rapidly growing throughout the world during the past few decades^2,3^. PTC patients diagnosed at late stages have a five-year survival rate <60%, and the recurrence has been reported to be as high as 30%^4^. Hence, there is an urgent need for identifying more specific and sensitive biomarkers to explorate the pathogenesis of PTC. These include the telomerase reverse transcriptase promoter region (*TP53*, *BRAF*, and *RAS*) as well as other gene mutations that can be used in the exploration of the pathogenesis of thyroid cancer. Molecular markers and their related molecular pathways of genetic and epigenetic changes can also be helpful in developing targeted therapies^5^, so identifying PTC-related molecular markers is important for exploration of the pathogenesis of PTC.

Tumour related biomarkers have a variety of forms, including pathological biomarkers, epigenetic biomarkers, protein biomarkers, DNA biomarkers, and RNA biomarkers. The mRNAs that play a key role in the protein translation process can also be used as biomarkers for exploration of the pathogenesis of cancer^6^. Garcia and colleagues reported that the level of cyclin D1 mRNA in plasma can be used as a possible marker of clinical outcomes in breast cancer^7^, and March-Villalba reported that hTERT mRNA was a useful noninvasive tumour marker for the molecular diagnosis of prostate cancer^8^.

For the detection of mRNA levels, the most commonly used methods include northern blots, the polymerase chain reaction (PCR), RNA *in situ* hybridization, cDNA microarrays, and high-throughput sequencing techniques. RNA sequencing (RNA-Seq) has become a widely-accepted method for detection of gene expression levels^9^. It provides a more comprehensive method for mapping and quantifying transcriptomes, when compared with gene chips or other sequencing techniques^10,11^. Although the data obtained by RNA-Seq is massive, bioinformatics can analyse the large data comprehensively, systematically, and accurately. It is therefore possible to identify key elements or genes associated with human disease from the high-throughput data obtained from this technique.

The development of bioinformation technology, the emergence of various public databases, and the application of analytical strategies have provided powerful tools for the analysis and identification of differentially expressed genes. The GO database is currently the most widely-used gene annotation system for gene functions and products^12^. It can perform functional enrichment analyses of target genes, and provide a better understanding of the relationships between genes and diseases. The KEGG database combines genetic information with functional information, and can be used to systematically analyse the relationships between gene functions and enriched pathways^13^. Protein-protein interaction (PPI) network analysis is also widely used in data processing. It can intuitively analyse the interactions between proteins, in order to accurately assess the interaction between genes^14^.

In this study, we performed RNA-Seq and utilized bioinformatics technology to identify genes that were differentially expressed genes (DEGs) in stage I PTC tissues vs. matched normal adjacent tissues. The Cancer Genome Atlas database and qt-PCR were used for double validation. The relationship interactions between DEGs were analysed by protein-protein interaction networks and chromosome localizations. Finally, four newly discovered genes, *COMP*, *COL3A1*, *ZAP70*, and *CD247*, were found to be related with PTC clinical phenotypes, and were confirmed by Spearman’s correlation analyses in TCGA database. The expression level of *COMP* was significantly and positively related to the tumour sizes of PTC patients. The higher the gene expression, the larger the tumour size. In addition, the expression levels of *COL3A1*, *COMP*, and *ZAP70* were positively related to the risk of lymph node metastasis. Furthermore, *COL3A1* and *COMP* expression levels were correlated with the TNM stage in PTC patients. These four DEGs might be promising biomarkers for early-stage PTC, and provide an experimental foundation for further exploration of the pathogenesis of early-stage PTC.

## Results

### Screening of DEGs based on RNA-Seq

In order to discover novel genes related to the pathogenesis of papillary thyroid carcinoma (PTC) by using differentially expressed genes (DEGs), we selected the female patients with stage I PTC. First of all, three patients who met the above criteria were enrolled in this study. It was a reasonable amount of patients to do the initial RNA-Seq experiments^15,16^. Therefore, the three pairs of stage I PTC tissues and matched normal adjacent tissues were sequenced by RNA-Seq. Then we tried to gather samples as much as possible, 17 patients who met the above criteria were also enrolled in this study. But four patients’ tissues were unable to carry out qRT-PCR because of RNA degradation. So 13 patients were enrolled in this study. In total, 16 patients were enrolled in this study (Table 1).

**Table 1:** Clinical information on 16 PTC patients.

Number	gender	age	tumor diameter (cm)	TNM	Stage
1	female	41	1.00	T_1_N_1_M_0_	Stage I
2	female	39	1.30	T_1_N_1_M_0_	Stage I
3	female	37	2.00	T_1_N_1_M_0_	Stage I
4	female	38	1.40	T_1_N_1_M_0_	Stage I
5	female	57	1.20	T_1_N_0_M_0_	Stage I
6	female	45	0.80	T_1_N_0_M_0_	Stage I
7	female	41	1.50	T_1_N_0_M_0_	Stage I
8	female	42	2.00	T_1_N_1_M_0_	Stage I
9	female	29	1.50	T_1_N_1_M_0_	Stage I
10	female	42	1.20	T_1_N_0_M_0_	Stage I
11	female	47	0.80	T_1_N_0_M_0_	Stage I
12	female	54	0.90	T_1_N_0_M_0_	Stage I
13	female	61	1.80	T_1_N_0_M_0_	Stage I
14	female	26	1.20	T_1_N_0_M_0_	Stage I
15	female	49	0.70	T_1_N_0_M_0_	Stage I
16	female	37	2.00	T_4_N_1_M_0_	Stage I

**Figure 1:**
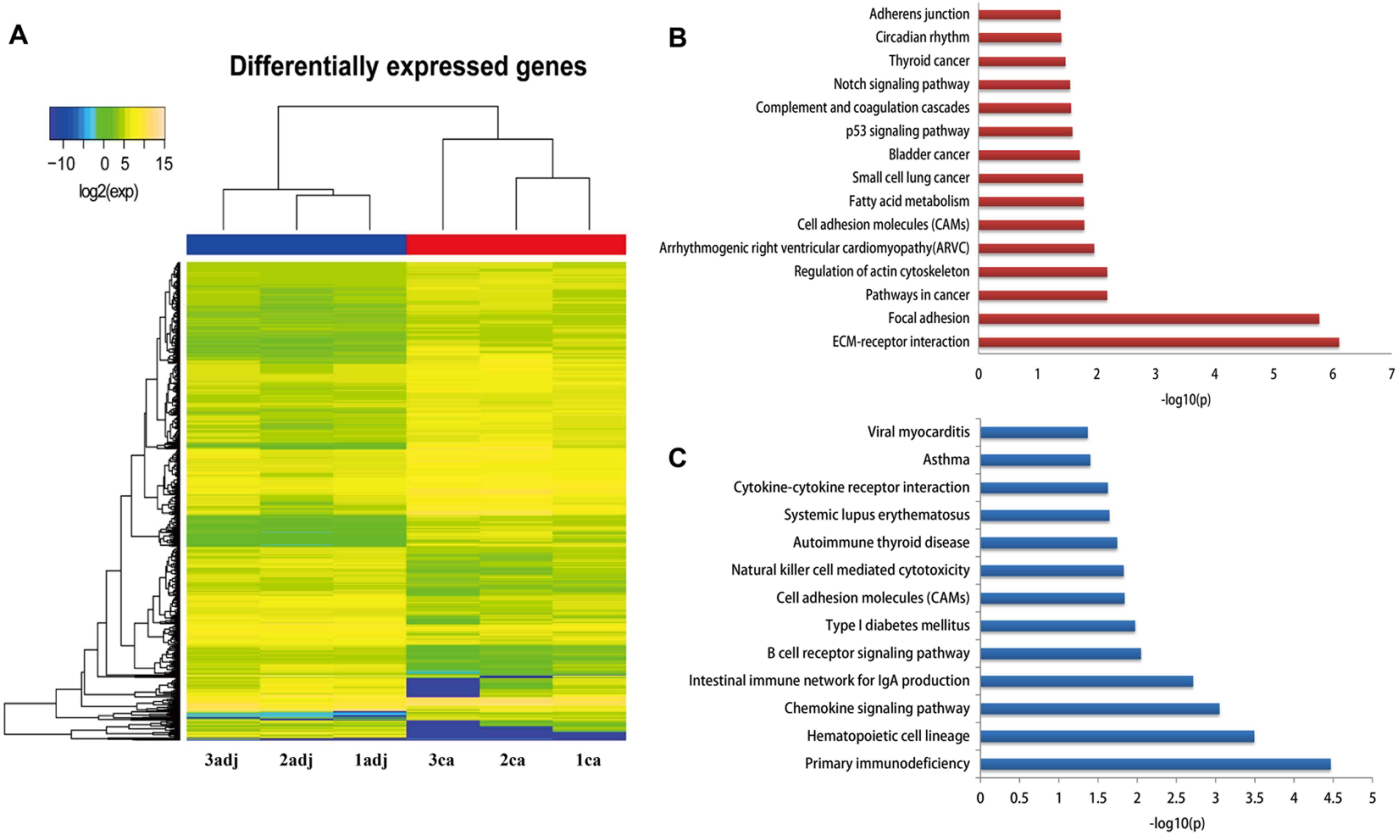
Hierarchical clustering and significantly enriched KEGG pathways of differentially expressed genes. (**A**) Numbers, the sample number; ca, cancer tissue; adj, adjacent normal tissue; and exp, gene expression values. The expression level for each gene is represented by a colour range from blue (low) to yellow (high). (**B**) Significantly-enriched KEGG pathways of upregulated genes. (**C**) Significantly enriched KEGG pathways of downregulated genes.

**Figure 2:**
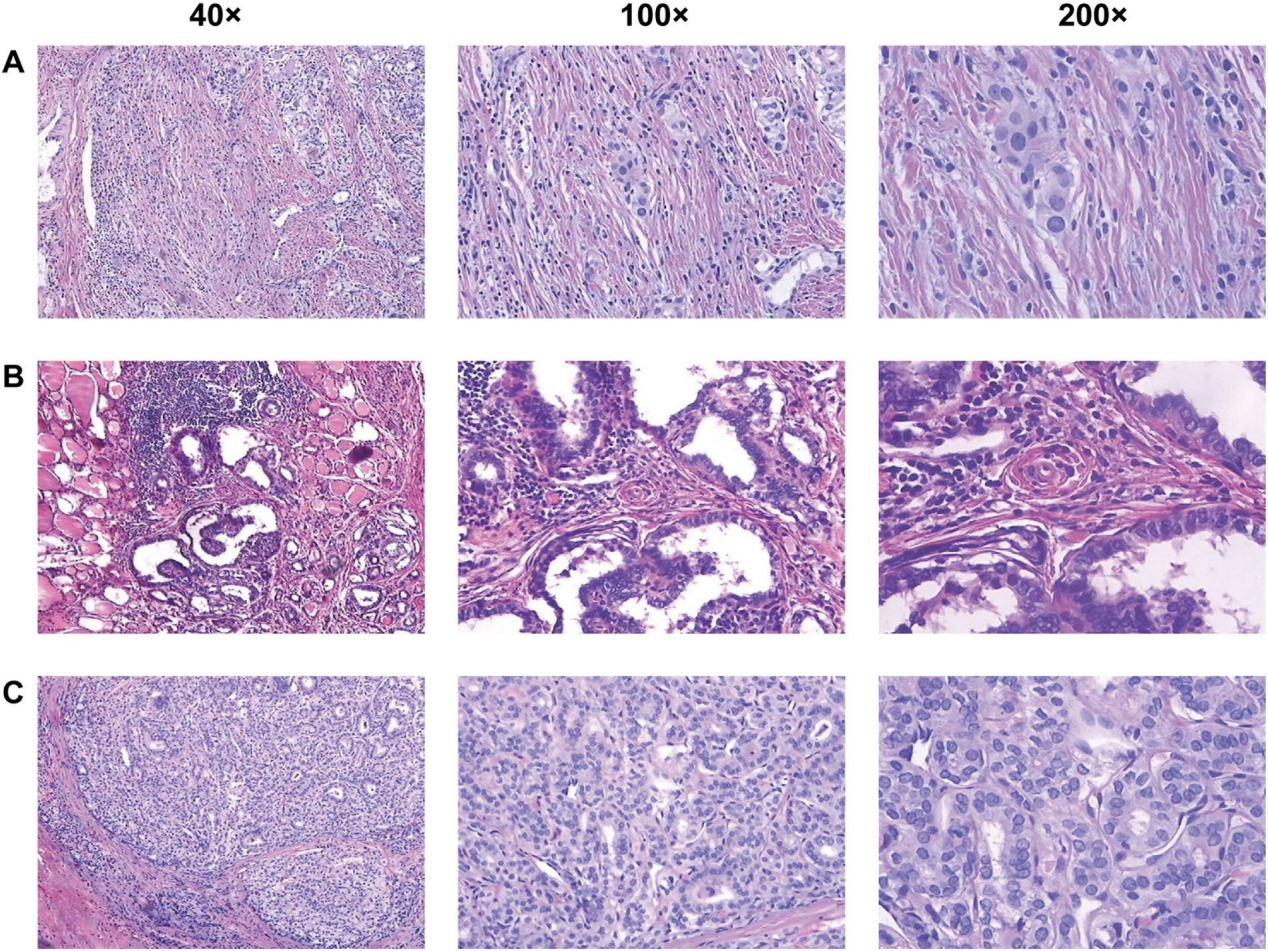
Haematoxylin and eosin staining for papillary thyroid carcinoma (PTC) tissues. (**A**–**C**) Represent the three samples of PTC; 40×, 40 times the visual field observation; 100×, 100 times the visual field observation; and 200×, 200 times the visual field observation.

We obtained 9–11 million reads for each sample after RNA sequencing (RNA-Seq). A total of 13,703 unique genes were detected by removing the genes with transcript per million mapped (RPKM) values <0.5 from the analyses. We calculated the difference of RPKM values and the fold changes between cancer samples and matched normal adjacent samples. A difference of a RPKM value >10 and a fold change >1.5 were used to classify the DEGs. Based on this definition, there were 456 upregulated [Eca(i) Eadj(i) > 1.5 i = 1,2,3] and 263 downregulated [Eadj(i)/Eca(i) > 1.5 i = 1, 2, 3] genes. These 719 DEGs were regarded as candidate genes for further study, and their expression levels are shown in a heat map in Fig. 1A. The results of haematoxylin and eosin (HE) staining of the three pairs of stage I PTC tissues are shown in Fig. 2.

### KEGG signalling pathway enrichment analysis

To investigate the DEG-related pathways to reveal potential mechanisms of PTC, we performed enrichment analyses to identify related pathways. The 456 upregulated genes were highly enriched in 15 pathways and the 263 downregulated genes in 13 pathways (p < 0.05). Significantly enriched KEGG pathways are shown in Fig. 1B,C. The lower the p value, the more significant the enrichment. KEGG analyses indicated that the upregulated, DEGs were involved in multiple tumorigenesis pathways, including pathways in thyroid cancer, small cell lung cancer, bladder cancer, the p53 signalling pathway, cell adhesion molecules, focal adhesion, adherens junctions, and extracellular matrix (ECM)-receptor interactions. Downregulated genes were significantly involved in KEGG pathways related to autoimmune thyroid disease, natural killer cell-mediated cytotoxicity, cytokine-cytokine receptor interactions, chemokine signalling pathways, B-cell receptor signalling pathways, intestinal immune networks for IgA production, systemic lupus erythematosus, viral myocarditis, asthma, and type I diabetes mellitus.

### Integrated KEGG pathway regulatory networks

To further screen DEGs and identify the relationships between genes and diseases, we extracted the relationship between all genes in 28 KEGG pathways enriched by the DEGs, and constructed an integrated KEGG pathway regulation network.

**Figure 3:**
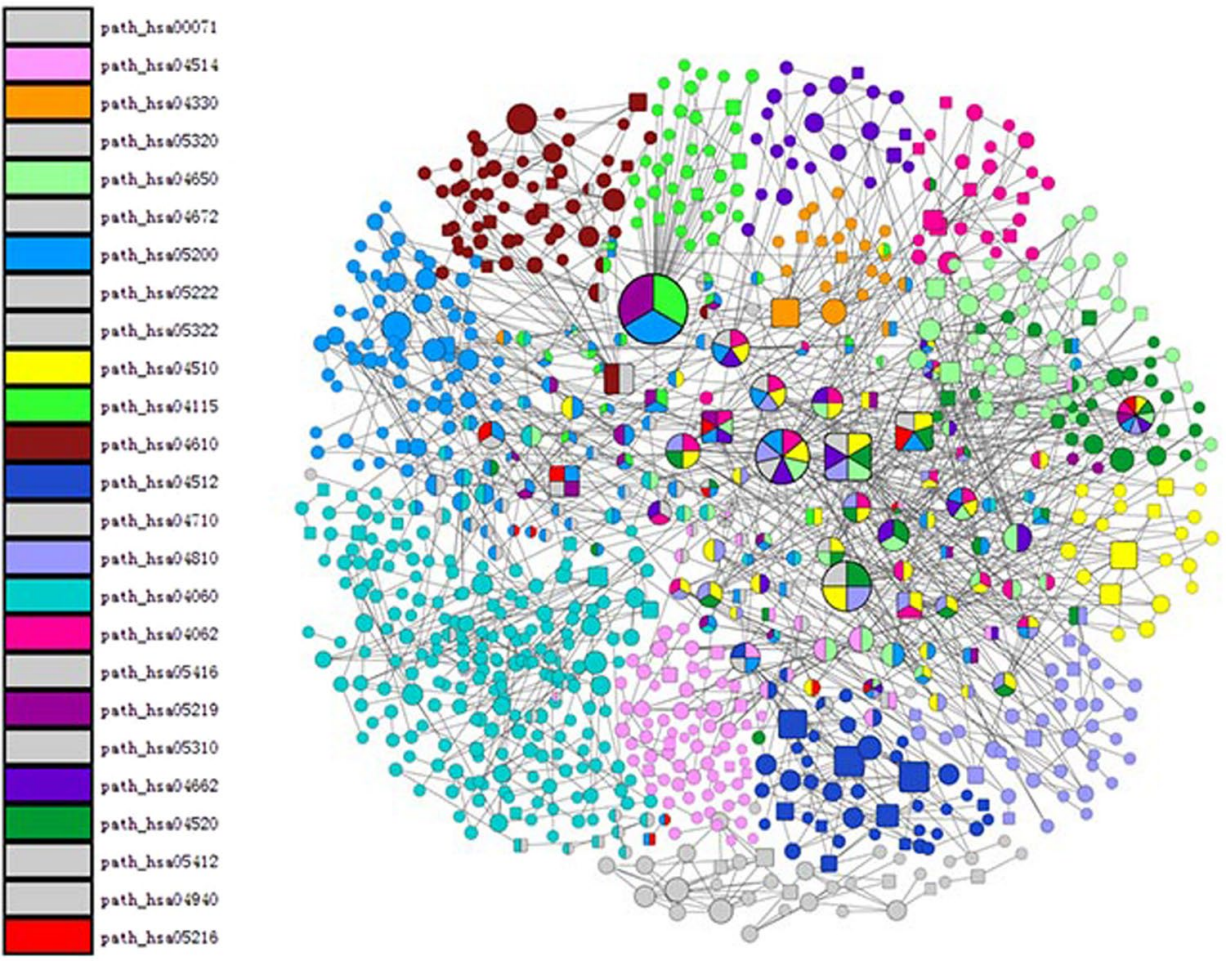
The integrated KEGG pathways regulatory network. Different colours represent different pathways; square nodes represent the nodes that included the differentially expressed genes (DEGs), the round nodes represent nodes that did not include the DEGs.

The network included 857 nodes and 1,224 edges. Each node in the network represented a data object in KEGG, which was the product of one or more genes. The edges represented the relationships. Different colours represented different pathways. There were ten pathways with less nodes, so we represented them uniformly with grey. If a node was comprised of different colours, the node appeared in different pathways. The square nodes represented nodes included in the DEGs, while the rounded nodes represented those not included in the DEGs. The size of the nodes represented the degrees of node distributions. The larger the node, the higher the degree (Fig. 3).

Because the network was too large to obtain additional important information, we chose nodes whose degrees were in the top 10% of the networks, which contained the differentially expressed genes. A total of 18 nodes containing 16 upregulated genes and four downregulated genes were identified in the networks (Supplementary Table S1). The upregulated genes included *CTNNB1*, *HRAS*, *FN1*, *CCND1*, *C3*, *LAMA5*, *LAMB1*, *LAMB3*, *COL1A1*, *COL3A1*, *NOTCH4*, *ITGB4*, *PXN*, *COMP*, *CDKN1A*, and *ITGA3*. The downregulated genes included *RAC2*, *ZAP70*, *IL2RG*, and *CD247*.

### GO functional enrichment analysis

**Figure 4:**
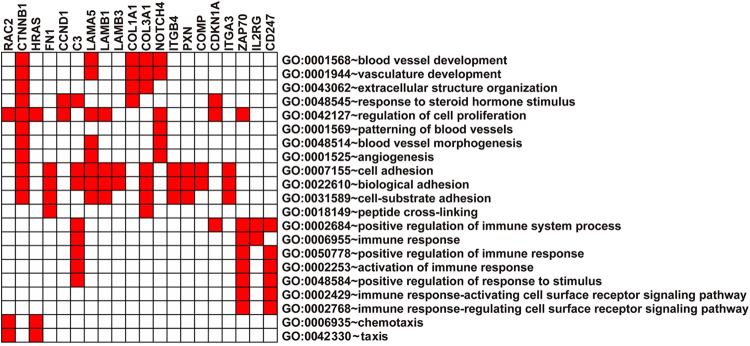
Functional enrichment analysis of 20 differentially expressed genes. Red boxes represent the significant enrichment of GO terms.

Functional enrichment analyses were used to further investigate functional differences of the 20 differentially expressed genes in the integrated KEGG pathway regulatory networks. From the GO results, 21 biology processes were significantly overrepresented for 20 differentially expressed genes (adjusted values of p < 0.05). The enrichment terms are shown in Fig. 4. The following differentially expressed genes were significantly enriched in GO: 0007155 ~ cell adhesion (11 genes) and GO: 0022610 ~ biological adhesion (11 genes).

### Validation of DEGs by qRT-PCR and TCGA database

**Figure 5:**
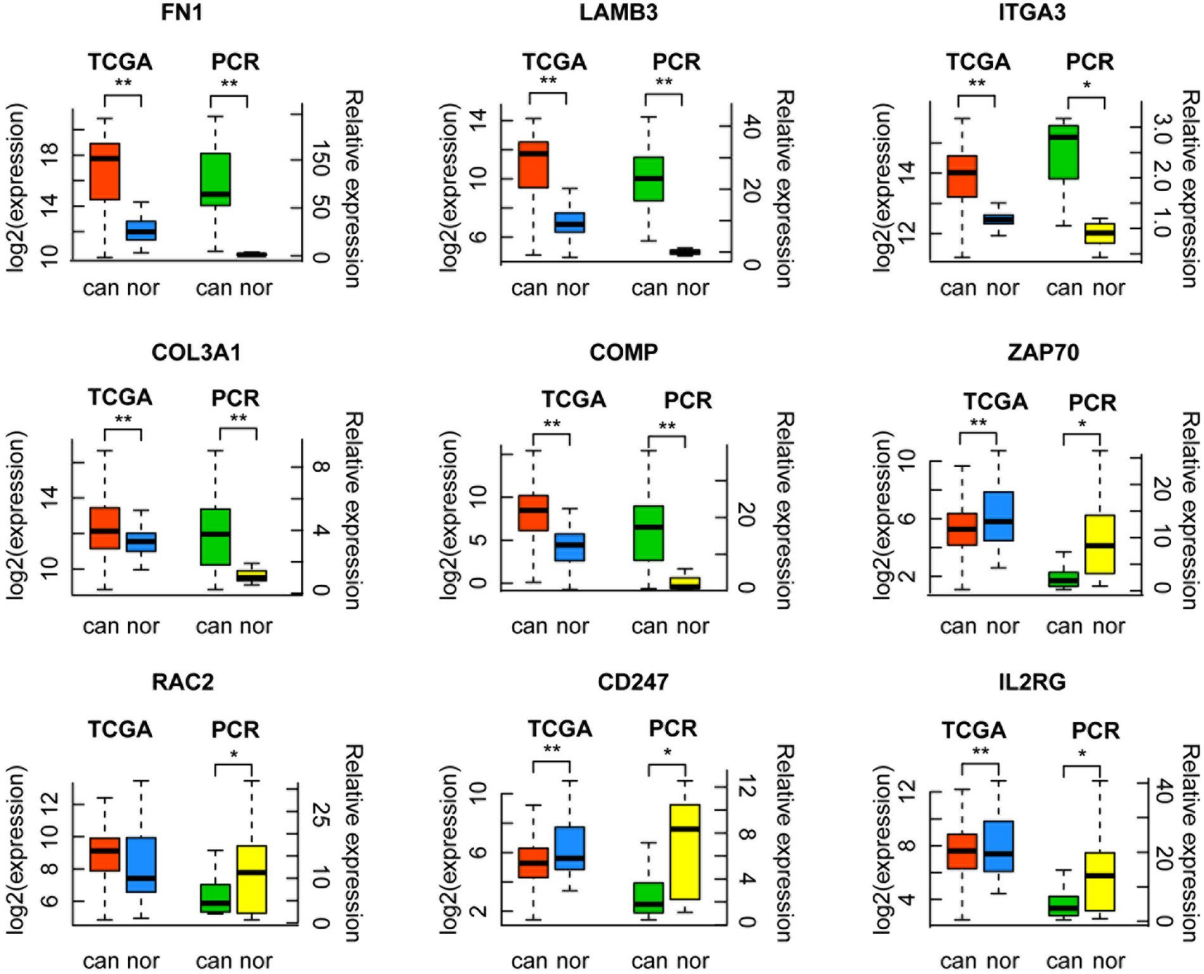
qRT-PCR and TCGA verification results of nine genes. TCGA, TCGA database validation results; PCR, qRT-PCR validation results; can, cancer tissue; nor, adjacent normal tissue *p < 0.05; **p < 0.01.

In the 20 selected DEGs, ten genes (*COMP*, *COL3A1*, *LAMA5*, *LAMB1*, *PXN*, *C3*, *RAC2*, *ZAP70*, *IL2RG*, and *CD247*) were first found to be associated with PTC in this study, and were not previously reported. The other ten genes were specifically reported to be associated with PTC^17–26^. All newly discovered PTC-related genes were selected to be validated. And from those ten genes which were reported to be associated with PTC, we randomly selected three PTC-related genes (*FN1*, *ITGA3*, *and LAMB3*). We then used qRT-PCR to validate the mRNA levels of 13 DEGs in stage I PTC tissues and matched normal adjacent tissues from 16 stage I PTC patients. Among the selected 13 genes, nine genes showed consistent expression differences with the RNA-Seq data, and the three genes, which were reported to be associated with PTC, were all validated. Among the identified genes, *COMP* (p = 0.0002), *COL3A1* (p = 0.0026), *FN1* (p < 0.0001), *ITGA3* (p = 0.0112), *LAMB3* (p = 0.0005) levels were increased in stage I PTC tissues. In contrast, the expressions of *RAC2* (p = 0.0405), *ZAP70* (p = 0.0121), *IL2RG* (p = 0.0175) and *CD247* (p = 0.0112) were downregulated in tumour tissues (Fig. 5). TCGA gene expression data by RNA sequencing from 513 PTC samples and 59 normal samples were used as the validation cohort, and 4,137 DEGs were screened out. The results showed that among the nine genes verified by qRT-PCR, seven genes (*COMP*, *COL3A1*, *FN1*, *ITGA3*, *LAMB3*, *ZAP70*, and *CD247*) were validated and consistent with the RNA-Seq and qRT-PCR data. The three genes, which were reported to be associated with PTC, were all validated (Fig. 5).

### PPI networks and chromosome locations

**Figure 6:**
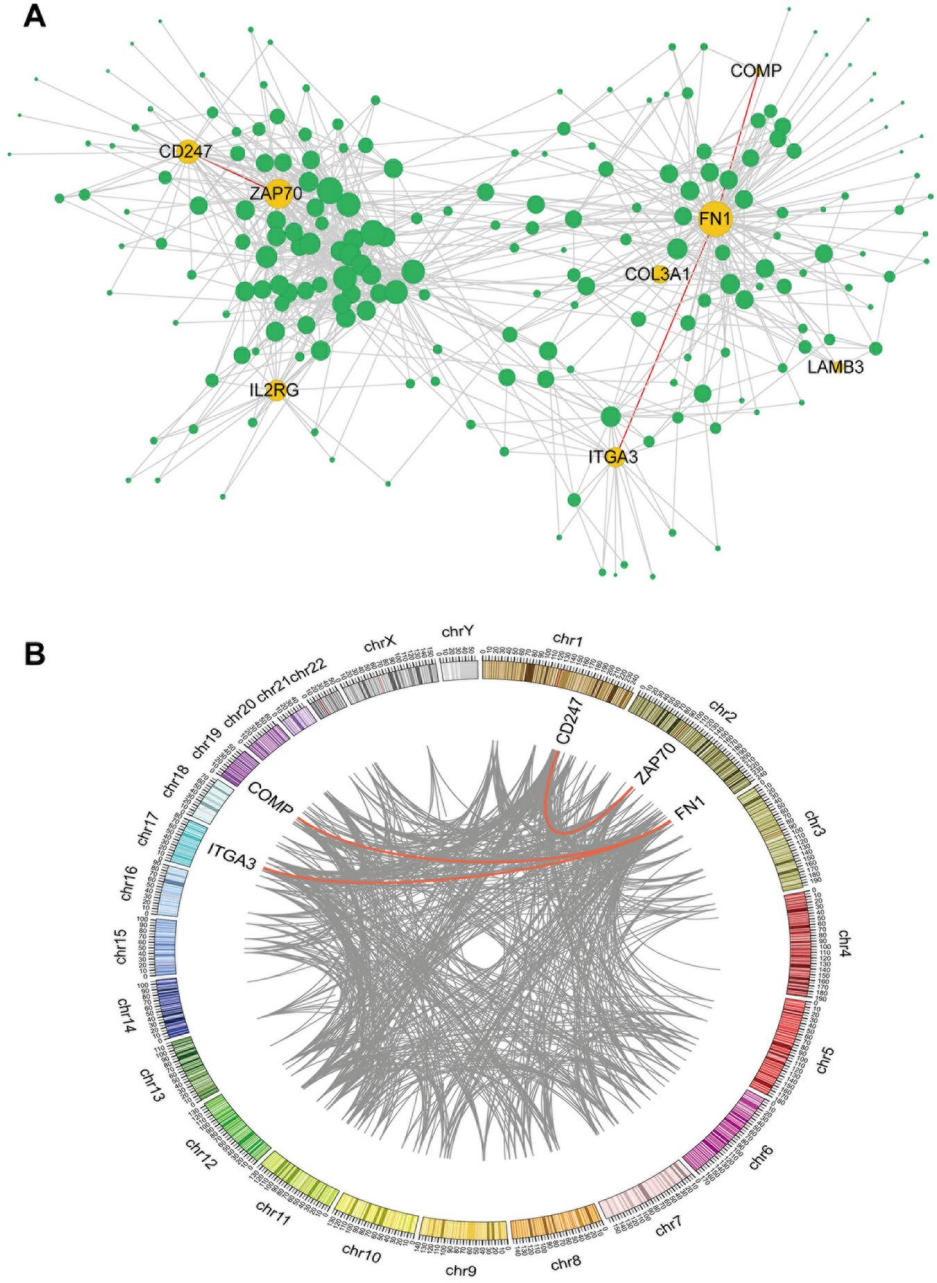
The Protein-Protein interaction (PPI) network and the distribution of the interacted genes on chromosomes. (**A**) The yellow dots represent the identified seven differentially expressed genes (DEGs); the green dots represent the genes interacted with the seven DEGs; the lines represent PPIs. (**B**) Different colours represent different chromosomes; the scale marked on each chromosome represents the genetic map distance. The lines represent PPIs; red lines represent the relationship between the identified three pairs of DEGs; the rest of the interacting relationship is shown with grey lines.

To clarify the interactions between the verified DEGs and to identify the intrinsic mechanism of the genes and diseases, we constructed the PPI network and analysed the protein interactions between the seven genes (Fig. 6). There were three pairs of interacting genes, *FN1* and *COMP*, *FN1* and *ITGA3*, and *CD247* and *ZAP70*. Using the Ensembl database chromosome positioning and Circos mapping of the three pairs of genes, we determined that the three pairs of genes were located on different chromosomes (Fig. 6).

### Correlation between the DEGs and clinical characteristics of PTC

To determine the correlation between the identified DEGs and PTC clinical characteristics, a total of 504 PTC samples with clinical phenotypic data in TCGA database were included. The correlations between the DEG expression levels and clinical characteristics [tumour size, lymph nodes metastasis, distant metastasis, and tumour node metastasis (TNM) staging] were analysed using Spearman’s correlation, with Gu’s method used as a ref.^27^. A value of p < 0.05 was defined as indicating a statistical significance. As shown in Table 2, the expression level of *COMP* was significantly and positively related to the tumour sizes of PTC patients. The higher the gene expression, the larger the tumour size. In addition, the expression levels of *COL3A1*, *COMP*, and *ZAP70* were positively related to the risk of lymph node metastasis. Furthermore, *COL3A1* and *COMP* expression levels were correlated with the TNM stage in PTC patients. In addition, we used *t*-test to analyse the correlation between the DEG expression levels and distant metastasis. We subdivided the gene expression levels of *CD247*, *ZAP70*, *COMP*, and *COL3A1* into two groups based on whether they had distant metastasis or not, respectively. The results showed that there was no significant difference between the two groups (P = 0.755, 0.76, 0.837, 0.306, respectively).

**Table 2:** Correlation analysis between 4 identified DEGs and clinical characteristics of PTC.

Gene		Tumor size	Lymph node	Metastasis	TNM stage
CD247	r	−0.033	0.089	−0.088	−0.073
	*P*	0.462	0.058	0.137	0.102
COL3A1	r	−0.014	0.151**	0.079	−0.112*
	*P*	0.755	**0**.**001**	0.179	**0**.**012**
COMP	r	0.132**	0.261**	−0.041	0.107*
	*P*	**0**.**003**	**1**.**72** × **10**^**−8**^	0.487	**0**.**016**
ZAP70	r	−0.036	0.115*	−0.048	−0.066
	*P*	0.427	**0**.**014**	0.414	0.138

r: spearman correlation; *P*: Sig.(2-tailed); *P < 0.05; **P < 0.01.

## Discussion

Papillary thyroid carcinoma (PTC) was the only histological type of tumour with incidence rates rising consistently among all ethnic groups over the past three decades^28^. Thus, it is important to identify appropriate biomarkers for exploration of the pathogenesis of this cancer.

We identified 719 differentially expressed genes (DEGs), with KEGG pathway enrichment analyses showing that the upregulated genes were significantly enriched in the pathways related to focal adhesions, ECM-receptor interactions, adherens junctions, and 12 other pathways. These pathways were all closely related to cancer. Focal adhesions are large protein complexes linking the cell cytoskeleton with the ECM. They affect many cellular processes including motility, proliferation, differentiation, regulation of gene expression, and cell survival^29^. Moreover, this pathway has been found to be significantly associated with gene expression studies in many types of cancers^30–32^.

To further screen DEGs and explore the relationships between genes and diseases, we constructed the integrated KEGG pathway regulation network. We chose the DEGs in the nodes whose degrees were in the top 10% in the network. A total of 20 DEGs were identified. GO functional enrichment analyses were then used to further investigate the function of these 20 DEGs, indicating that they were significantly enriched in 21 GO terms closely related to cancer, such as regulation of cell proliferation, cell adhesion, biological adhesion, and cell substrate adhesion.

To confirm the reliability of our methods and the experimental data, we screened the DEGs related to stage I PTC, and validated the selected 13 DEGs by qRT-PCR and TCGA databases. The positive results were further analysed using the PPI network. Knowledge of the PPI network helps to solve many problems such as signalling pathways identification^33,34^, recognition of functional modules^35^, and prediction of protein functions^36^. We can therefore assess the interactions between DEGs and understand the intrinsic mechanisms between genes and diseases more accurately. Among the selected 13 genes, nine genes showed consistent expression differences with the RNA-Seq data, and the three genes, which were reported to be associated with PTC, were all validated. Among the identified genes, *COMP* (p = 0.0002), *COL3A1* (p = 0.0026), *FN1* (p < 0.0001), *ITGA3* (p = 0.0112), *LAMB3* (p = 0.0005) levels were increased in stage I PTC tissues. In contrast, the expressions of *RAC2* (p = 0.0405), *ZAP70* (p = 0.0121), *IL2RG* (p = 0.0175) and *CD247* (p = 0.0112) were downregulated in tumour tissues. TCGA gene expression data by RNA sequencing from 513 PTC samples and 59 normal samples were used as the validation cohort, and 4,137 DEGs were screened out. The results showed that among the nine genes verified by qRT-PCR, seven genes (*COMP*, *COL3A1*, *FN1*, *ITGA3*, *LAMB3*, *ZAP70*, and *CD247*) were validated and consistent with the RNA-Seq and qRT-PCR data. The three genes, which were reported to be associated with PTC, were all validated, and the high positive percentages confirmed the reliability of our studies. Among the seven gene products, *FN1* and *COMP*, *FN1* and *ITGA3*, and *CD247* and *ZAP70* interacted with each other. Cancer is a complex disease caused by the interaction of multiple environmental factors and genes^37^. Gene expression is a complex and orderly process that is regulated by cis-acting elements and trans-acting factors^38^. Regulation of gene expression at the level of transcription is often associated with trans-acting proteins and cis-acting promoter sequences that work together to affect the function of RNA polymerase (RNAP). In response to environmental cues, regulatory proteins can interact directly with RNAP to alter its activity or interact with specific sequences or structures in the promoter region to affect RNAP binding or processing. Certain genes are subject to complex controls involving multiple trans-acting factors and sequences in the promoter region in order to function co-ordinately or independently to affect transcription^39^. In our study, the above three pairs of interacting genes were located on different chromosomes, suggesting that the three pairs of genes affected the expression of each other through trans-acting and protein interactions, which then affected the occurrence and development of PTC.

In our study, *COL3A1* (collagen, Type III, alpha 1) and *COMP* (cartilage oligomeric matrix protein), were significantly upregulated in stage I PTC tissues (*COL3A1*, p = 0.0026; *COMP*, p = 0.0002). Both of the genes were co-enriched in GO terms of cell adhesion, biological adhesion, and cell substrate adhesion. Cell adhesion is involved in stimulating signals that regulate cell differentiation, cell cycle, cell migration, and cell survival. Tumour cells are characterized by changes in the adhesion to the ECM, which may be related to their invasive and metastatic potentials^40^. Moreover, *COL3A1* was related to blood vessel development and vasculature development. It is well-known that tumour growth and metastasis are complementary processes. When tumour cells are switched to an angiogenic phenotype, tumour growth and progression occur^41^. In our study, there was a notable correlation between *COL3A1* and lymph node metastasis. Su *et al*. suggested that *COL3A1* may increase renal cell carcinoma growth, metastasis, and tumour macrophage infiltration^42^. Another study reported that high *COL3A1* mRNA and/or protein expression was accompanied with a high stage, as well as smoking and the recurrence of colorectal cancer^43^. Using analyses of the PPI network, we found that *COMP* and *FN1* interacted with each other, and had similar GO functions. We found that *COMP* was positively correlated with tumour size, lymph node metastasis, and TNM stage. In addition, in a study of prostate cancer^44^, breast cancer^45^, and other cancers, *COMP* was also found to be closely related to tumorigenesis. All these findings indicated that the upregulation of *COL3A1* and *COMP* is closely related to the occurrence and development of cancer. However, the identification of *COL3A1* and *COMP* as potential therapeutic targets or molecular markers of PTC still require a more complete understanding of their mechanisms of action.

*ZAP70* (zeta-chain associated protein kinase; 70 kDa) and *CD247* (*CD247* molecule) were both downregulated in PTC tissues. They were co-enriched in many GO terms of immune response. It is well-known that there is a close connection between inflammation and cancer. Autoimmune diseases always result in tissue destruction and inflammation, or even an increased risk of PTC^46^. In addition, an interaction between the *ZAP70* and *CD247* genes was found using PPI analysis. The two genes were co-enriched in natural killer cell-mediated cytotoxicity in the KEGG pathway. Once activated, NK cells are able to reserve large amounts of cytotoxic granules containing perforin and granzymes that produce cytotoxicity of tumour cells^47^. Thus, downregulation of *ZAP70* and *CD247* expression in this pathway may lead to an attenuation of NK cell-mediated cytotoxicity of the tumour, which in turn leads to the occurrence of PTC. We also found that *ZAP70* was positively correlated with lymph node metastasis, so it may be related to the invasion and metastasis of PTC.

In conclusion, four DEGs, *COMP*, *COL3A1*, *ZAP70*, and *CD247*, were identified by RNA-Seq and bioinformatic methods. *ZAP70* and *CD247* were co-enriched in many immune response-related functions, and *COMP* and *COL3A1* were associated with cell adhesion and biological adhesion during the development of PTC. *FN1* and *COMP*, *FN1* and *ITGA3*, and *CD247* and *ZAP70* interactions may influence the expression of each other by trans-acting and protein-protein interactions, which in turn may affect the development of PTC. The expression level of *COMP* was significantly and positively related to the tumour sizes of PTC patients. The higher the gene expression, the larger the tumour size. In addition, the expression levels of *COL3A1*, *COMP*, and *ZAP70* were positively related to the risk of lymph node metastasis. Furthermore, *COL3A1* and *COMP* expression levels were correlated with the TNM stage in PTC patients. These four DEGs might be promising biomarkers for early-stage PTC, and provide an experimental foundation for further exploration of the pathogenesis of early-stage PTC.

## Materials and Methods

### Patients and tissue procurement

In order to discover novel genes related to the pathogenesis of papillary thyroid carcinoma (PTC) by using differentially expressed genes (DEGs), we selected the female patients with stage I PTC. First of all, three patients who met the above criteria were enrolled in this study. It was a reasonable amount of patients to do the initial RNA-Seq experiments^15,16^. Therefore, the three pairs of stage I PTC tissues and matched normal adjacent tissues were sequenced by RNA-Seq. Then we tried to gather samples as much as possible, 17 patients who met the above criteria were also enrolled in this study. But four patients’ tissues were unable to carry out qRT-PCR because of RNA degradation. So 13 patients were enrolled in this study. In total, 16 patients were enrolled in this study. These 16 stage I PTC patients underwent thyroidectomy at The Second Affiliated Hospital of Harbin Medical University (China) from November of 2013 to January of 2014. The entire cohort consisted of 16 females, with a mean age of 42.8 ± 9.3 years. According to the Union for International Cancer Control and the American Joint Committee on Cancer on Tumour Node Metastasis classification, all these patients presented as TNM stage I. We obtained stage I PTC tissues and matched normal adjacent tissues that were >2 cm from the tumour without infiltration. All tissues were obtained at the time of surgical resection, then immediately frozen in liquid nitrogen and stored at −80 °C. Clinical and histopathological information was collected for all patients. All methods were performed in accordance with the relevant guidelines of the ethics committee of The Second Affiliated Hospital of Harbin Medical University, and all patients granted informed consent. The experimental protocols were approved by the ethics committee of The Second Affiliated Hospital of Harbin Medical University.

### Haematoxylin and eosin (HE) staining

HE staining was used to assess the sections of three sequenced stage I PTC tissues. After deparaffinization and rehydration, 5 μm longitudinal sections were stained with hematoxylin solution for 5 min followed by 5 dips in 1% acid ethanol (1% HCl in 70% ethanol) and then rinsed in distilled water. Then the sections were stained with eosin solution for 3 min and followed by dehydration with graded alcohol and clearing in xylene. The mounted slides were then examined and photographed using an Olympus BX53 fluorescence microscope (Tokyo, Japan).

### RNA extraction, library preparation, and sequencing

Total RNA was extracted from stage I PTC tissues and matched normal adjacent tissues of 16 stage I PTC patients, using TRIzol reagent (Qiagen, Valencia, CA, USA) according to the manufacturer’s instructions. Total RNA was then stored at −80 °C until used. RNA quantity and purity were assessed by using a Nanodrop (OD 260/280 ratio). RNAs of stage I PTC tissues and matched normal adjacent tissues from three stage I PTC patients (sample number: 1ca, 1adj, 2ca, 2adj, 3ca, and 3adj; the number denoted different samples, the “ca” denoted a cancer sample, and the “adj” denoted a matched, normal, adjacent sample) were used. Six libraries were constructed using an Illumina standard kit according to the manufacturer’s protocol. All sequencing was performed on an Illumina Hiseq. 2500 instrument. The RNA-Seq reads involved 8–11 million with 101 nt unique ends.

### Data analysis

#### Read mapping and differentially expressed genes analyses

Read mapping and differentially expressed genes screening TopHat^48^ (version 2.0.6) were used to map RNA-Seq reads to reference genomes (Ensembl Human Genome GCRh37). Parameters with default values were used. Following mapping of the sequencing reads, the transcripts were assembled with Cufflinks^49^ (version 2.2.1). Then, the cuff norm was used to quantify the expression levels for each gene normalized by reads per kb of RPKM reads (1). RPKM ≥ 0.5 was defined as a mapped gene. The mapped genes were then used to calculate the difference of RPKM values and the fold changes between cancer samples and matched normal adjacent samples. A difference >10 and a fold change >1.5 were classified as a DEG.

1 $${\rm{RPKM}}=\frac{{\rm{total}}\,{\rm{exon}}\,{\rm{reads}}}{{\rm{mapped}}\,{\rm{reads}}\,({\rm{millions}})\times {\rm{exon}}\,{\rm{length}}\,({\rm{KB}})}$$

#### Functional enrichment analysis

The functional enrichment analyses for differentially expressed genes were performed using the DAVID function annotation tool (http://david.abcc.ncifcrf.gov/home.jsp), which included the KEGG pathway, biological processes, molecular functions, and cellular components. A value of p < 0.05 was defined as significant enrichment.

#### Construction of the KEGG pathway integrated network

The integrated network of the KEGG pathway was constructed. The relationships between DEGs in significant enrichment in the KEGG pathways were extracted in R (http://www.r-project.org) with the XML package (R, version 2.15.2, Bioconductor, version 2.3). The network was visualized using Cytoscape^50^.

#### GO functional enrichment analysis

GO is a standard classification system of gene function and gene products^12^. We chose the DEGs in the nodes whose degrees were in the top 10% in the network. The GO terms with Benjamini-adjusted p < 0.05 in DAVID were used.

### Data validation

#### qRT-PCR validation

In the abovementioned selected DEGs, all newly discovered PTC-related genes were selected to be validated. And from those ten genes which were reported to be associated with PTC, we randomly selected three PTC-related genes. Validations of the mRNA levels of DEGs were performed using quantitative real-time PCR (qRT-PCR). Total RNAs were extracted as mentioned above from stage I PTC tissues and matched normal adjacent tissues of 16 stage I PTC patients. The cDNAs were synthesized using a first strand cDNA synthesis kit (Takara RR036A; Takara, Japan) according to the manufacturer’s instructions. Subsequently, 1 μL of cDNA product and each gene specific primer were used for PCR, using the Real-time PCR Master Mix kit (Takara RR820A), which was implemented using an ECO fluorescence quantitative PCR system (Illumina, USA). Relative gene expression values were calculated using the 2^−ΔΔCt^ method^51^.

#### TCGA database validation

To make our results more reliable, we downloaded thyroid cancer RNA-Seq V2 isoform expression profiles of 513 PTC samples and 59 normal samples from TCGA to validate the positive DEGs that qRT-PCR had verified. The R package, “edgeR,” was used and the genes with values of p < 0.05, fold change >1.5 (or <2/3) between tumour and adjacent normal samples were validated.

### Analysis of the PPI networks and chromosomal locations

We chose the integrated PPI network as background, which was integrated from the Biomolecular Interaction Network Database, the Biological General Repository for Interaction Data sets, the Database of Interacting Proteins, the Human Protein Reference Database, IntAct, the Molecular IN Teraction database, the mammalian PPI database of the Munich Information Center on Protein Sequences, PDZBase (a PPI database for PDZ-domains), and Reactome. The validated DEGs of TCGA database were put into the background network of the PPI, and the protein interaction pairs were screened to construct the protein interaction subnet.

The chromosomal positions of genes that interacted with proteins were mapped using the Ensembl database. The mapping software, Circos, was used to identify the chromosomal location, and connected the interacted genes.

### Correlations between the DEGs and clinical characteristics of PTCs

To identify correlations between the identified DEGs and PTC clinical characteristics, a total of 504 PTC samples with clinical phenotypic data in TCGA database were included. The correlations between DEG expression levels and clinical characteristics (tumour size, lymph nodes metastasis, distant metastasis, and TNM staging) were analysed using Spearman’s correlation, with Gu’s method used as a ref.^27^. In addition, we used *t*-test to analyse the correlation between the DEG expression levels and distant metastasis. We subdivided the gene expression levels of *CD247*, *ZAP70*, *COMP*, and *COL3A1* into two groups based on whether they had distant metastasis or not, respectively. A value of p < 0.05 was defined as indicating significance.

## Electronic supplementary material

Supplementary information
