# Supplementary material for: Identification of Biomarkers Based on Differentially Expressed Genes in Papillary Thyroid Carcinoma
Source: Sci Rep. 2018 Jul 2;8:9912. doi: 10.1038/s41598-018-28299-9 (PMC6028435; doi:10.1038/s41598-018-28299-9)
Supplement: Supplementary file 1 — Supplementary information [file 41598_2018_28299_MOESM1_ESM.pdf]

Identification of Biomarkers Based on Differentially Expressed Genes in Papillary Thyroid Carcinoma

Jun Han[1#], Meijun Chen[1#], Yihan Wang[2], Boxuan Gong[3], Tianwei Zhuang[4], Lingyu Liang[5], Hong Qiao[1]*

[1]Department of Endoerinology and Metabolism, The Second Affiliated Hospital, Harbin Medical University, Harbin, 150001, China

[2]College of Bioinformatics Science and Technology, Harbin Medical University, Harbin, 150081, China

[3]Faculty of Vehicle Engineering and Mechanics, Dalian University of Technology, Dalian, 116024, China

[4]Department of Endoerinology and Metabolism, Mu danjiang Medical University Affiliated Hongqi Hospital, Mu danjiang, 157000, China

[5]Internal medicine, Hebei Provincial Eye Hospital, Xingtai, Hebei, 054001, China

[#]These authors have contributed equally to this work

*To whom correspondence should be addressed. Tel/Fax: 08686297720; Email: qiaoh0823@sina.com

Supplementary Table

Supplementary Table S1: The top 10% nodes in the network

Degree	Pathway name	Gene symbol
24	Focal adhesion; Adherens junction; Natural killer cell mediated cytotoxicity; Regulation of actin cytoskeleton; B cell receptor signaling pathway; Viral myocarditis	RAC1, RAC2*, RAC3
18	Focal adhesion; Adherens junction; Pathways in cancer; Thyroid cancer;	CTNNB1[#]

	Arrhythmogenic right ventricular cardiomyopathy	
14	Chemokine signaling pathway; Natural killer cell mediated cytotoxicity; B cell receptor signaling pathway; Pathways in cancer; Thyroid cancer; Bladder cancer	HRAS#, KRAS, NRAS
13	ECM-receptor interaction	FN1#
13	Pathways in cancer; Bladder cancer; Bladder cancer; Small cell lung cancer; Thyroid cancer	CCND1#
13	Complement and coagulation cascades; Systemic lupus erythematosus	C3#
13	ECM-receptor interaction	LAMC3, LAMB4, LAMA1, LAMA2, LAMA3, LAMA4, LAMA5#, LAMB1#, LAMB2, LAMB3#, LAMC1, LAMC2
12	ECM-receptor interaction	COL1A1#, COL1A2, COL2A1, COL3A1#, COL4A1, COL4A2, COL4A3, COL4A4, COL4A5, COL4A6, COL5A1, COL5A2, COL6A1, COL6A2, COL6A3, COL11A1, COL11A2, COL6A6, COL24A1, COL6A5, COL5A3, COL27A1
12	Notch signaling pathway	NOTCH1, NOTCH2, NOTCH3, NOTCH4#

11	Focal adhesion	ITGB1, ITGB3, ITGB4#, ITGB5, ITGB6, ITGB7, ITGB8
10	Focal adhesion; Chemokine signaling pathway; Regulation of actin cytoskeleton	PXN#
8	ECM-receptor interaction	COMP#, THBS1, THB2, THBS3, THBS4
8	p53 signaling pathway; Thyroid cancer; Pathways in cancer; Bladder cancer	CDKN1A#
7	Focal adhesion	ITGA11, ITGA6, ITGA1, ITGA2, ITGA2B, ITGA3#, ITGA4, ITGA5, ITGA7, ITGA9, ITGAV, ITGA10, ITGA8
7	Natural killer cell mediated cytotoxicity	ZAP70*
6	Chemokine signaling pathway	RAC1, RAC2*
6	Cytokine-cytokine receptor interaction	IL2RG*
6	Natural killer cell mediated cytotoxicity	CD247*

#：Up-regulated genes；*：Down-regulated genes
